# Magnitude and Determinants of Under-Nutrition Among Late Adolescent Girls in East Africa: Evidence From Demographic and Health Surveys (2010–2016)

**DOI:** 10.3389/fnut.2022.763047

**Published:** 2022-04-04

**Authors:** Temam Beshir Raru, Galana Mamo Ayana, Mohammed Abdurke Kure, Bedasa Taye Merga, Mohammed Yuya, Kedir Teji Rob

**Affiliations:** ^1^School of Public Health, College of Health and Medical Sciences, Haramaya University, Harar, Ethiopia; ^2^School of Nursing and Midwifery, College of Health and Medical Sciences, Haramaya University, Harar, Ethiopia

**Keywords:** under-nutrition, late adolescent, girls, East Africa, DHS

## Abstract

**Background:**

Adolescent girls are vulnerable to undernutrition owing to their increased nutrition demand for growth and development, including sexual development and maturation. Despite its public health importance, undernutrition among late adolescent girls has received little attention in health and nutrition policies. Although undernutrition in adolescent girls has been investigated, most of the previous study reports were based on small sample sizes and limited geographic settings. Therefore, we aimed to estimate the prevalence and determinants of undernutrition among late adolescent girls in East Africa.

**Methods:**

Secondary data analysis was conducted among 10 East African countries using the most recent Demographic and Health Survey (DHS) reports. A total of 21,779 adolescent girls aged 15–19 years were included in this study. Descriptive statistics were conducted to describe the study population. The binary logistic regression model was fitted to identify the determinants of undernutrition among late adolescent girls. Variables with a *p*-value of <0.05 in the multivariate analysis were identified as statistically significant determinants of undernutrition.

**Results:**

The overall magnitude of undernutrition among late adolescent girls in East Africa was 16.50% (95% CI: 16.00–17.00), whereas the overall magnitude of obesity among late adolescent girls in East Africa was 2.41% (95% CI: 2.21–2.62). In the final model of the multivariate analysis, adolescent girls aged 18–19 years [adjusted odds ratio (AOR) = 0.55; 95% CI: 0.51–0.60], having secondary education (AOR = 0.79; 95% CI: 0.68–0.93), being from a rich wealth index family (AOR = 0.63; 95% CI: 0.56–0.69), and being from a medium wealth index family (AOR = 0.80; 95% CI: 0.72–0.89) were negatively and statistically associated with undernutrition. Likewise, having more than seven household members (AOR = 1.36; 95% CI: 1.10–1.67), walking more than 30 min to a water source (AOR = 1.10; 95% CI: 1.01–1.20), and living in Ethiopia (AOR = 1.75; 95% CI: 1.51–2.03) were positive determinants of undernutrition among late adolescent girls.

**Conclusion:**

In this study, undernutrition in late adolescent girls remains a considerable public health problem in East Africa. Age group, educational status, marital status, family wealth index, family size, time taken to reach a water source, media exposure, and country of resident were significant determinants of undernutrition. Therefore, devising strategies that improve the socioeconomic status of households and/or adolescents would help to reduce the risks of undernutrition in late adolescents.

## Introduction

The period of transition in which a child matures into an adult is called adolescence (1) and female adolescents aged 15–19 years are defined as late adolescent girls (2). In general, when compared with under-five children and older adults, adolescents are supposed to enjoy good health and are less prone to infection and, thus, usually receive little health and nutritional care except for concerns regarding reproductive health (2). Due to increased nutrition demand for growth and development, including sexual development, maturation, and the initiation of menarche, adolescent girls are vulnerable to undernutrition (3). Adolescent girls aged 15–19 years are more impacted by undernutrition in low- and middle-income countries (2).

Undernutrition among adolescent girls is a major public health concern all over the world (4, 5). (2) In developing countries, undernutrition among late adolescent females is far worse and remains a major public health problem; however, it has received less policy attention. The highest burden of undernutrition among adolescent girls in the world was concentrated in the South Asian and sub-Saharan Africa (SSA) regions (2). For instance, in South Asia, about two out of five adolescent girls are undernourished (6). Similarly, a secondary analysis of 11 Asian and SSA countries revealed the highest prevalence of undernutrition among adolescent girls in Nigeria (32.9%) (7). Similarly, East African countries have a higher burden of undernutrition in late adolescent girls (2).

Furthermore, undernutrition is responsible for poor development and health outcomes, including poor educational achievement, loss in productivity, and increased risk of infectious and non-infectious diseases (8). Studies have shown that undernourished adolescent girls are mostly malnourished during their childhood and infancy period and in the future they will continue to become malnourished mothers, giving birth to babies with low birth weight, which tends to become an intergeneration cycle (9, 10). Moreover, stillbirths, small-for-gestational-age neonates, complicated delivery, and even maternal death may be attributed to undernutrition during the adolescence period (11).

Different studies have shown that age, educational status, occupation, latrine type, and place of residence were significantly associated with undernutrition (12–14). Scholars also stated that family size, dietary diversity, absence of latrine, the unprotected water source for drinking, and food-insecure households were significant predictors of undernutrition among late adolescent girls (12, 15, 16).

Even though the sustainable development goals (SDGs) include an adolescent nutrition service which is addressing adolescent malnutrition, the nutritional status of adolescent girls is not improving in most of the African countries (17). To track the progress of health-related targets of SDGs, having evidence about the pooled prevalence and determinants of undernutrition among late adolescent girls at the regional level has paramount importance. However, there is limited evidence in East African countries about the magnitude and determinants of undernutrition among late adolescent girls. In addition, most of the previous studies were based on small sample sizes and were conducted in a limited geographic setting. Therefore, this study aimed to estimate the magnitude and determinants of undernutrition among late adolescent girls in 10 East African countries using the most recent Demographic and Health Survey (DHS) data from 2010 to 2016.

## Materials and Methods

### Data Source, Tools, and Sampling Procedure

**FIGURE 1:**
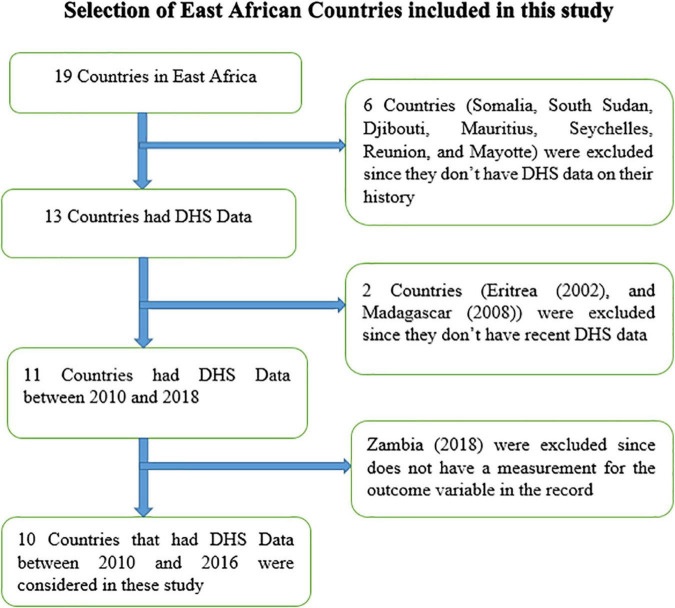
Schematic diagram for the selection of East African countries included in the analysis of DHS data from 2010 to 2016.

This study was a secondary data analysis based on the DHS (1). The data were obtained from the Measure DHS program^1^ after authorization was granted through an online request by explaining the goal of this study. The DHS program adopts standardized methods involving uniform questionnaires, manuals, and field procedures to gather information comparable across countries globally. The DHSs are nationally representative household surveys that provide data from a wide range of monitoring and impact evaluation indicators in population, health, and nutrition with face-to-face interviews of women aged 15–49. The surveys employ a stratified, multistage, and random sampling design. Information was obtained from eligible women aged 15–49 years in each country (18). Detailed survey techniques and methods of sampling used to collect data have been recorded elsewhere (19). In this study, the DHS data were pooled from the 10 East African countries from 2010 to 2016. We used the recent DHS of country-specific dataset during the specified period in the individual record dataset and extracted the dependent and independent variables. The 10 East African countries from which data were extracted are Burundi, Comoros, Ethiopia, Kenya, Malawi, Mozambique, Rwanda, Tanzania, Uganda, and Zimbabwe (Table 1). All the late adolescent girls in the 10 East African countries who have a measurement for the outcome variable were included. A total of 21,779 late adolescent girls were included in the analysis of this study. The samples included from each country were listed below (Table 1). According to the United Nations, there are 19 countries in East Africa. In history, only 13 countries had the DHS data, 2 countries (Eritrea and Madagascar) had no recent DHS data, and one country (Zambia) had no record for the outcome variable, i.e., undernutrition, which was left null on the survey, and 10 countries were included in this study (Figure 1).

**TABLE 1:** The demographic and health survey (DHS) years of study and study participants of undernutrition in the 10 East African countries from 2010 to 2016.

East African countries	DHS year	Number of participants
Burundi	2010	1,971
Comoros	2012	1,241
Ethiopia	2016	3,262
Kenya	2014	2,774
Malawi	2015/16	1,721
Mozambique	2011	3,031
Rwanda	2014/15	1,393
Tanzania	2015/16	2,905
Uganda	2011	1,381
Zimbabwe	2013/14	2,100
**Total Sample Size**		**21,779**

### Population and Eligibility Criteria

The source population for this study was all the non-pregnant and non-postpartum adolescent girls aged 15–19 years in East Africa. All the non-pregnant and non-postpartum adolescent girls aged 15–19 years in East Africa who were in the selected countries were the study population. Specific adolescent girls, who have no measurement for the outcome interest, were excluded from this study.

### Measurements

The outcome variable for this study was late adolescent girls’ undernutrition. It was measured with body mass index (BMI), which is a binary outcome variable and it is coded as 1 if the women undernourished (had BMI < 18.5 kg/m^2^) and 0 otherwise. Based on different studies of literature, independent variables considered in this study were age, marital status, residence, educational status, number of household members, number of under-five children, sex of household head, age of household head, wealth index, covered by health insurance, source of drinking water, time to get to a water source, type of latrine, media exposure, and living country.

Height and weight measurements are taken using a Shorr Board and SECA digital scale, respectively (20). The weight was recorded in kilograms (kg), accurate to 0.1 kg and the height was recorded in centimeters (cm), accurate to 0.1 cm. Finally, the BMI was generated from the weight and height of adolescents after converting the height to a meter (m).


B⁢M⁢I=W⁢e⁢i⁢g⁢h⁢t⁢(k⁢g)/(H⁢e⁢i⁢g⁢h⁢t⁢(m)*H⁢e⁢i⁢g⁢h⁢t⁢(m))

**Undernutrition:** It is a nutritional status, which is classified as either stunting or underweight (thinness) if the BMI is less than 18.5 kg/m^2^ (21).

**Total underweight (thinness):** It is defined as both acute and chronic malnutrition status that is measured by BMI < 18.5 kg/m^2^ (2).

**Normal BMI:** It is defined as well-nourished individuals with BMI 18.5–24.9 kg/m^2^ (2).

**Overnutrient (obesity):** It is defined as overnourished individuals with BMI ≥ 30 kg/m^2^ (2).

**Media exposure** was defined as when a woman reads a newspaper or listens to the radio, or watches television at least three times per week.

### Data Processing and Management

Data processing and analysis were performed using STATA 15 software. After extraction, the data were further cleaned, recoded, labeled, and weighted using sampling weight, primary sampling unit, and strata before any statistical analysis to restore the representativeness of the survey and to tell the STATA to take into account the sampling design when calculating SEs to get reliable statistical estimates.

### Statistical Analysis

Cross-tabulations and summary statistics were conducted to describe the study population. Simple frequencies, summary measures, tables, and figures were used to present the data. Binary logistic regression was used to assess relationships between the measures of undernutrition and independent variables. All the bivariable analyses yielding a *p*-value of ≤0.25 were retained for the multivariable model (22, 23). Variables with a *p*-value of <0.05 in multivariable analysis were identified as statistically significant predictors of undernutrition. Multicollinearity was assessed using the variance inflation factor (VIF) and considered no multicollinearity if lower than 10. An odds ratio with 95%CI was used to measure the strength of the associations. The goodness of fit of the binary logistic regression models was also checked using a Hosmer and Lemeshow test (24).

## Results

### Sociodemographic and Economic Characteristics of the Study Participants

A total of 21,779 adolescent girls aged 15–19 years in each country’s DHS survey were included in this study. More than third-fifth of the adolescent girls in East African countries lie in the age group of 15–17 years. The majority (71.92%) of the girls from East African countries were rural residents. Regarding the family wealth index of the adolescent girls in East African countries, 34.78% of them have a poor wealth index and 91.07% of the study populations were covered by health insurance. For 36.95% of the household in East African countries, it takes 30 min or longer to reach the water source. The majority of the respondents have no exposure to mass media (Table 2).

**TABLE 2:** Sociodemographic and economic characteristics of 10 East African countries from 2010 to 2016.

Variables	Category	Unweighted frequency (%)	Weighted frequency (%)
Undernutrition	Not under-nourished	17,927(82.31)	18,008(83.50)
	Under-nourished	3,852(17.69)	3,557(16.50)
Age	15–17	13,435(61.69)	13,240(61.39)
	18–19	8,344(38.31)	8,326(38.61)
Residence	Urban	7,137(32.77)	6,055(28.08)
	Rural	14,642(67.23)	15,510(71.92)
Education	No education	1,519(6.97)	1,416(6.57)
	Primary	10,915(50.12)	11,236(52.10)
	Secondary	9,119(41.87)	8,665(40.18)
	Higher	226(1.04)	248(1.15)
Current marital status	Never married	17,155(78.77)	16,840(78.09)
	Married	2,907(13.35)	2,966(13.76)
	Living with partner	1,195(5.49)	1,172(5.44)
	Widowed	12(0.06)	10(0.05)
	Divorced	216(0.99)	231(1.07)
	Separated	294(1.35)	345(1.60)
Family wealth index	Poor	7,498(34.43)	7,501(34.78)
	Medium	3,889(17.86)	4,206(19.50)
	Rich	10,392(47.72)	9,859(45.72)
Sex of house hold head	Male	14,041(64.47)	14,171(65.71)
	Female	7,738(35.53)	7,395(34.29)
Age of household head	15–24	2,308(10.60)	2,393(11.10)
	25–34	2,529(11.61)	2,518(11.68)
	35–44	5,139(23.60)	5,049(23.41)
	45–59	7,701(35.36)	7,463(34.61)
	≥60	4,102(18.83)	4,142(19.21)
Number of house hold member	1–2	1,611(7.40)	1,686(7.82)
	3–5	7,778(35.71)	7,778(36.07)
	6–7	5,826(26.75)	5,953(27.61)
	>7	6,564(30.14)	6,146(28.50)
Number under-five children	No under-five child	9,611(44.13)	9,586(44.45)
	At least one under-five child	12,168(55.87)	11,979(55.55)
Health insurance	No	19,914(91.44)	19,640(91.07)
	Yes	1,865(8.56)	1,925(8.93)
Source of drinking water	Protected	12,807(58.80)	12,263(56.87)
	Un-protected	8,106(37.22)	8,469(39.27)
	Others	866(3.98)	833(3.87)
Time to water source	Less than 30 min	6,822(31.32)	7,115(32.99)
	30 min or longer	7,654(35.14)	7,967(36.95)
	Water on premises	6,387(29.33)	5,608(26.00)
	Others	916(4.21)	875(4.06)
Type of toilet	Improved	10,026(46.04)	9,424(43.70)
	Not-improved	10,953(50.29)	11,365(52.70)
	Others	800(3.67)	776(3.60)
Media exposure	No	13,396(61.51)	13,549(62.82)
	Yes	8,383(38.49)	8,017(37.18)
Living country	Burundi	1,971(9.05)	1,910(8.86)
	Comoros	1,241(5.70)	1,254(5.81)
	Ethiopia	3,262(14.98)	3,222(14.94)
	Kenya	2,774(12.74)	2,625(12.17)
	Malawi	1,721(7.90)	1,729(8.02)
	Mozambique	3,031(13.92)	3,032(14.06)
	Rwanda	1,393(6.40)	1,389(6.44)
	Tanzania	2,905(13.34)	2,882(13.36)
	Uganda	1,381(6.34)	1,372(6.36)
	Zimbabwe	2,100(9.64)	2,148(9.96)

### Magnitude of Undernutrition

**FIGURE 2:**
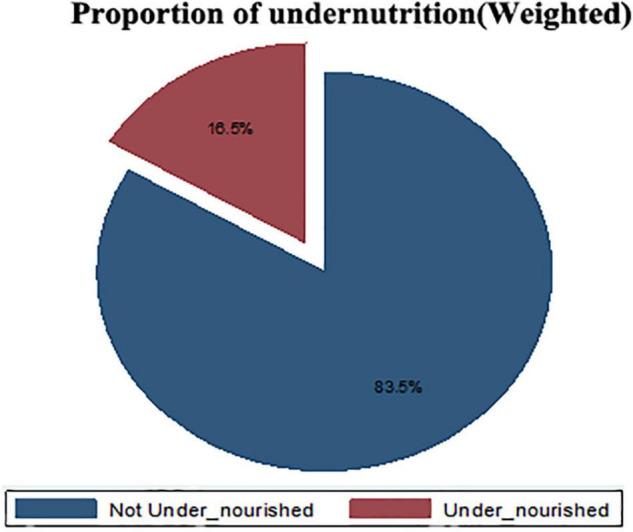
The overall magnitude of under-nutrition among late Adolescents in East African countries from 2010 to 2016.

The majority of the adolescent girls were included from Ethiopia and the minority were from Comoros. The overall magnitudes of undernutrition in East African countries was 16.50% (95% CI: 16.00–17.00), whereas the overall magnitude of obesity (overnutrition) in East African countries was 2.41% (95% CI: 2.21–2.62) (Figure 2).

### Overall Proportion of Nutritional Status Among Each Country

**FIGURE 3:**
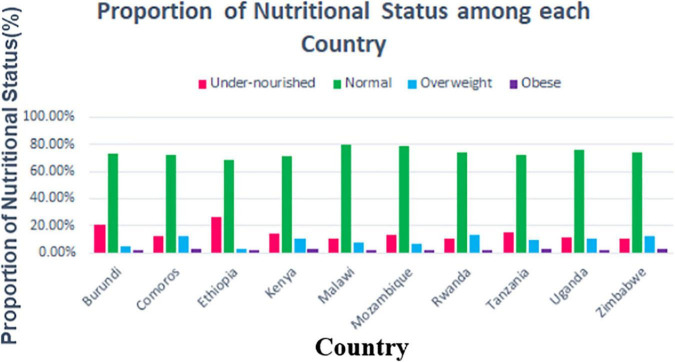
Proportion of nutritional status among adolescent girls in East African Countries from 2010 to 2016.

In this study, the highest and lowest magnitude of undernourishment was from Ethiopia (26.68%) (95% CI: 25.18–28.23) and Rwanda (10.13%) (95% CI: 8.64–11.82), respectively, while the highest and lowest magnitude of obesity was from Zimbabwe (3.48%) (95% CI: 2.78–4.34) and Burundi (1.64%) (95% CI:1.16–2.32), respectively (Figure 3).

### Determinants of Undernutrition

The results from the multivariable model reveal that age of adolescent girls, secondary education, current marital status, family wealth index, greater than or equal to 60 age of household head, number of household members, 30 min or longer time to a water source, media exposure, and country of resident were significantly associated with undernutrition in East Africa at 5% level of significance.

This study revealed that the odds of undernourishment were reduced by 45% among adolescent girls in the age group of 18–19 [adjusted odds ratio (AOR) = 0.55; 95% CI: 0.51–0.60] compared to the adolescent girl in the age group of 15–17. The odds of undernourishment were decreased by 21% among the adolescent girls who have secondary education than those who had no formal education (AOR = 0.79; 95% CI: 0.68–0.93). Adolescent girls from the rich family wealth index have reduced the risk of undernourishment by 37% as compared to those who are from the poor family wealth index (AOR = 0.63; 95% CI: 0.56–0.69). Moreover, for those adolescent girls found in households that had more than 7 members, the odds of undernourishment increased by 36%, compared to adolescent girls living in households that had 1–2 members (AOR = 1.36; 95% CI: 1.10–1.67). Regarding time to the water source, for the adolescent girls living with the household that travels 30 min or longer to the water source, the odds of undernourishment increased by 10%, compared to those who travel less than 30 min (AOR = 1.10; 95% CI: 1.01–1.20). The odd of undernourishment increased by 75% among adolescent girls living in Ethiopia, compared to those living in Burundi (AOR = 1.75; 95% CI: 1.51–2.03) (Table 3).

**TABLE 3:** Determinants of undernutrition among late adolescent girls in East African countries (2010–2016).

Variables		Undernutrition		COR(95%CI)	AOR(95%CI)
		Not under-nourished	Under-nourished		
Age	15–17	10,537	2,703	1	1
	18–19	7,471	854	0.48(0.45–0.52) **	0.55(0.51–0.60) **
Residence	Urban	5,319	736	1	1
	Rural	12,689	2,821	1.49(1.37–1.61) **	1.03(0.93–1.15)
Educational status of girls	No education	1,147	269	1	1
	Primary	9,072	2,164	0.77(0.68–0.88)**	0.95(0.83–1.09)
	Secondary	7,585	1,080	0.49(0.43–0.56)**	0.79(0.68–0.93)*
	Higher	204	44	0.60(0.42–0.87)*	1.12(0.75–1.66)
Marital status	Never married	13,768	3,072	1	1
	Married	2,642	324	0.64(0.57–0.72) **	0.59(0.51–0.69) **
	Living with partner	1,085	86	0.34(0.27–0.42) **	0.45(0.35–0.56) **
	Widowed	8	4	2.09(0.63–6.94)	2.04(0.59–7.08)
	Divorced	194	36	0.86(0.60–1.23)	0.66(0.45–0.96)
	Separated	311	34	0.37(0.24–0.56) **	0.47(0.30–0.72) **
Family wealth index	Poor	6,021	1,479	1	1
	Medium	3,447	759	0.74(0.67–0.82) **	0.80(0.72–0.89) **
	Rich	8,539	1,320	0.55(0.51–0.59)**	0.63(0.56–0.69) **
Sex of house hold head	Male	11,741	2,430	1	1
	Female	6,267	1,128	0.92(0.86–0.99) *	0.95(0.87–1.02)
Age of house hold head	15–24	2,138	255	1	1
	25–34	2,186	332	1.14(0.97–1.34)	0.90(0.75–1.08)
	35–44	4,117	932	1.54(1.34–1.77) **	0.90(0.75–1.07)
	45–59	6,141	1,323	1.51(1.32–1.73) **	0.88(0.74–1.04)
	≥60	3,426	715	1.42(1.23–1.64) **	0.79(0.66–0.94) *
Family size	1–2	1,494	192	1	1
	3–5	6,607	1,173	1.34(1.14–1.57) **	1.24(1.04–1.48)
	6–7	4,895	1,058	1.57(1.34–1.85) **	1.29(1.06–1.57) *
	>7	5,012	1,134	1.67(1.42–1.96) **	1.36(1.10–1.67) **
Number under-five children	No under-five child	8,068	1,519	1	1
	At least one under-five child	9,940	2,039	1.07(0.99–1.15)	1.07(0.98–1.17)
Health insurance	No	16,381	3,259	1	1
	Yes	1,628	298	0.87(0.77–0.99)	1.08(0.94–1.26)
Source of drinking water	Protected	10,335	1,928	1	1
	Unprotected	6,942	1,527	1.27(1.18–1.36) **	1.05(0.96–1.15)
	Others	731	102	0.74(0.61–0.91) *	1.24(0.75–2.06)
Time to water source	Less than 30 min	5,912	1,202	1	1
	30 min or longer	6,459	1,508	1.24(1.14–1.34) **	1.10(1.01–1.20) *
	Water on premises	4,862	746	0.77(0.71–0.85) **	1.02(0.90–1.14)
	Others	774	101	0.61(0.49–0.75) **	0.62(0.37–1.04)
Type of toilet	Improved	8,237	1,188	1	1
	Not-improved	9,089	2,275	1.62(1.51–1.74) **	1.10(1.00–1.20)
	Others	682	94	0.85(0.68–1.06)	1.03(0.54–1.96)
Media exposure	No	11,010	2,538	1	1
	Yes	6,998	1,019	0.63(0.58–0.68) **	0.83(0.76–0.91) **
Living country	Burundi	1,487	422	1	1
	Comoros	1,094	159	0.52(0.43–0.64) **	0.62(0.50–0.77) **
	Ethiopia	2,317	905	1.61(1.41–1.83) **	1.75(1.51–2.03) **
	Kenya	2,208	417	0.89(0.77–1.02)	0.89(0.76–1.04)
	Malawi	1,527	202	0.42(0.35–0.51) **	0.46(0.37–0.56) **
	Mozambique	2,614	418	0.50(0.43–0.58) **	0.61(0.51–0.72) **
	Rwanda	1,242	146	0.41(0.33–0.50) **	0.42(0.34–0.52)^**^
	Tanzania	2,411	470	0.72(0.62–0.83) **	0.82(0.70–0.96)
	Uganda	1,20	163	0.52(0.43–0.63) **	0.51(0.41–0.62) **
	Zimbabwe	1,896	252	0.49(0.41–0.58) **	0.58(0.48–0.69) **

Key:** implies significant at p-value < 0.001 and * implies significant at p-value < 0.01.

***Crude odds ratio (COR)**: is just an odds ratio of **one independent variable** for predicting the **dependent variable**. **Adjusted odds ratio (AOR)**: holds other relevant variables constant and provides the odds ratio for the potential variable of interest which is adjusted for the other **independent variables** included in the model, i.e., controlling for confounding variables.*

## Discussion

In this study, the overall magnitude of undernutrition among late adolescent girls in 10 East African countries was 16.5% with the highest (4.74%) and the lowest (0.67%) magnitudes being from Ethiopia and Rwanda, respectively. Adolescent girls’ age, educational status, family wealth index, traveling greater than 30 min to reach the nearest water source, family size, media exposure, and country of residents were factors independently associated with undernutrition in late adolescent girls.

The proportion of undernutrition in late adolescent girls is relatively comparable with the previous secondary evidence from 57 low- and middle-income countries (15.6%) (24) and sub-Sahara African countries (15.14%) (25). It is also in line with a previous research report in Kenya (15.6%) (7), South Asia (19.8%) (24), and Ethiopia (17.3%) (26). However, the proportion of undernutrition reported in this study was higher compared to studies conducted in different parts of the world such as systematic review reports from Asian countries (8.8%) (27) and secondary analyses (28) of 54 developed and developing countries (8.4%) (29), 40 low- and middle-income African countries (6.30%) (30), and one systematic review and meta-analysis from Ethiopia (11.3%) (31). It is also much higher than studies conducted in the Gambia (12%) (32) and Ethiopia (thinness: 4.7% and stunting: 5.2%) (33). The possible justification for this discrepancy might be attributed to the geographical settings of the study population and the small sample size used in the specific study of the country, which may result in the underestimation of the problem. For instance, the previous lower proportion of undernutrition observed in the Gambia was country-based analysis and this might not be comparable with adolescents’ undernutrition in 10 pooled data of East African countries. In addition, the lower prevalence observed in the review analysis of Asian countries was because health service accessibility and exposure to information regarding dietary diversity might be better compared to this study population because the majority of our study participants were from rural and semiurban settings of East African countries.

In contrast, the result of this study is encouraging as the proportion of under-nutrition in late adolescent girls is much lower than the previous studies conducted elsewhere like 47.4% from secondary analysis of nutritional surveillance survey in Ethiopia (34), 36.5% from Myanmar (the DHS analysis from sub-Saharan and Asian countries) (24), 32.9% in Nigeria (secondary analysis of 11 Asian and SSA countries) (7), 25.7% in Nepal (35), 21.3% in Ethiopia (16), and in Assam, India (thinness: 25.7% and stunting: 31.33%) (36). The possible justification for these differences can be explained by the time gap between the study periods, geographical setting of the study population, and difference in methods and sample size of this study.

In the final model of the multivariate analysis, adolescents’ age was found to be an independent predictor of undernutrition. Thus, adolescent girls aged 18–19 years were 45% less likely to be undernourished compared to adolescent girls in the age group of 15–17 years. This is also supported by findings from DHS analysis of 38 developing countries (37), and secondary analysis of nutritional surveillance survey in Ethiopia (34). In addition, a similar association was also observed by studies conducted in India (13), Southern Ethiopia (38), and Indonesia (39). The possible explanation for this is because of a synergistic effect of growth velocity during puberty, variation in growth and development between the two age groups, and endocrine factors are also essential for promoting normal adolescent growth and are sensitive to undernutrition. Hence, if the requirement for achieving their maximum need for growth and development is not fulfilled, they will be prone to develop undernutrition (40).

Likewise, educational status was significantly associated with undernutrition in late adolescent girls. Accordingly, the risk of undernourishment was 0.79 times lower among the adolescent girls who had secondary education than those adolescents with no formal education. Similar reports were also observed in studies conducted by Berhe and Gebremariam, Soharwardi et al., and Teferi et al. (33, 37, 41). The possible explanation is that exposure to information about dietary diversity plays a paramount. This is because education could allow adolescent girls to have better choices and decisions of dietary intake than uneducated adolescent girls.

Moreover, in this study, the family wealth index was found to be an independent predictor of undernutrition in late adolescent girls. Thus, adolescent girls from the rich family wealth index had reduced risk of undernourishment by 37% as compared to those adolescents from the poor family wealth index. This is incongruent with previous research reports conducted elsewhere (37–39). The possible justifications might be attributed to better household food security status observed in higher wealth index families. This better family income may play a significant role in the reduction of risks of undernutrition in adolescent girls.

Similarly, the large family size was also significantly associated with undernutrition in late adolescent girls. Those adolescent girls whose family size was greater than seven were 1.36 times more likely to be undernourished compared to adolescent girls living in households of 1–2 family members. Similar findings were also observed in the previous studies report from secondary analysis of 35 SSA countries (42), systematic review, and meta-analysis conducted by Berhe, et al. (12). Similarly, having a large family size was also found to be a significant predictor of undernutrition in late adolescent girls in specific study settings conducted elsewhere (36, 43, 44).

Likewise, the status of media exposure was also found to be an independent predictor of undernutrition for adolescent girls in SSA. Thus, those adolescent girls who had a history of exposure to media were reduced the risk of undernourishment by 17% compared to their counterparts (adolescent girls who had no exposure). Similar reports were also observed in studies conducted by Kumar P et al. (45) and Branca F et al. (46). The possible reasons for this association may be because of better awareness and information gain regarding the importance of a variety of food items and frequency of feeding patterns among those with listening radio.

Furthermore, country of origin was significantly associated with undernourishment in late adolescent girls. Thus, being an Ethiopian increased the risk of undernutrition by 1.75 compared to residents from Burundi. This might be because of divergence to access of food security across countries due to climate changes, rainfall distributions, and soil degradation and erosion for crop production.

Finally, time to reach the nearest water resource was significantly associated with undernutrition in late adolescent girls in SSA. Those adolescent girls who travel 30 min or longer to the nearest water source were 1.10 times more likely to be undernourished than those who travel less than 30 min. The possible justification is because of traveling a long distance to access clean water may affect the feeding practices of the girls.

### Limitations of This Study

In this study, we analyzed the DHS data of 10 SSA countries to investigate the correlates of undernutrition among late adolescent girls. The study was nationally representative. Since it was secondary data analysis, we did not find some important variables, such as food security, variety of foods, and clinical-related variables, and variables such as husband’s educational status and occupational status were not consistently collected in each countries survey. In addition, we have not reported the specific outcome of undernutrition like wasting, stunting, and thinness, since undernutrition like wasting, stunting, and thinness were calculated based on standard Z-score which is more appropriate for under-five children rather than for adolescents.

## Conclusion

In this study, the overall prevalence of undernutrition in late adolescent girls was found to be 16.5%. This indicated that undernutrition in adolescent girls remains a considerable public health problem in East Africa. Age group, educational status, marital status, family wealth index, family size, time to a water source, media exposure, and living country were significantly associated with undernutrition. This result provides a cue to give due consideration for late adolescent girls to mitigate risks of undernutrition through various techniques. Improving the socioeconomic status of adolescent girls through economic empowerment, access to media, and education would help decrease the prevalence of undernutrition among adolescent girls. As the large family size is found to increase the risk of undernutrition, promoting family planning has paramount significance. Moreover, the government of each country should work on reducing the prevalence and risks of undernutrition in adolescent girls.

## Data Availability Statement

Publicly available datasets were analyzed in this study. This data can be found here: www.measuredhsprogram.com.

## Author Contributions

TR, GA, and KR conceived the project idea and designed the study. BM, MK, and MY facilitated the data collection, analyzed the data, and participated in the data interpretation. TR drafted the manuscript. All authors took part in writing the manuscript, reviewed the draft, and finally agreed on the journal to which the article has to be published and read and approved the final draft of the manuscript and also agreed to be accountable for all contents of the manuscript under any circumstances.

## Conflict of Interest

The authors declare that the research was conducted in the absence of any commercial or financial relationships that could be construed as a potential conflict of interest.

## Publisher’s Note

All claims expressed in this article are solely those of the authors and do not necessarily represent those of their affiliated organizations, or those of the publisher, the editors and the reviewers. Any product that may be evaluated in this article, or claim that may be made by its manufacturer, is not guaranteed or endorsed by the publisher.

## Footnotes

1
www.measuredhs.com
